# The impact of symmetry design of intangible cultural heritage souvenir on tourists’ aesthetic pleasure

**DOI:** 10.3389/fpsyg.2022.987716

**Published:** 2022-08-22

**Authors:** Yuqing Liu, Meiyi Chen, Qingsheng Wang

**Affiliations:** School of Management, Research Center for Management Innovation and Evaluation, Tianjin University of Commerce, Tianjin, China

**Keywords:** souvenir, intangible cultural heritage, symmetry design, aesthetic pleasure, authenticity

## Abstract

Souvenirs play an important role in tourism development. They act not only as mementos, enabling tourists to relive and retain the memory of a particular journey, but also as main income sources for tourism destinations and stakeholders. Many intangible cultural heritages (ICH) have been developed into souvenirs, especially products made by traditional craftsmanship. ICH souvenirs facilitate cultural value that is understandable to tourists, who appreciate the design of the ICH souvenirs and their contributions to a pleasure and memorable journey. Based on the theory of beauty and the preference-for-prototypes theory, this study explored how symmetry design of ICH souvenirs influences tourist’s aesthetic pleasure. As ICH souvenirs development is a commercialization process, and over-commodification would lead to cultures being lost and tourists’ disappointment, the authenticity concept is applied in order to address over-commodification. Thus, this study analyzed the moderating role of tourists’ authenticity perception of ICH souvenirs. Two lab-based between-subjects design experiments were employed to test the proposed hypotheses. Data analysis entailed multiple regression analysis, one-way ANOVA, and two-way ANOVA. The findings showed that symmetry of ICH souvenir design had a positive impact on tourists’ aesthetic pleasure. Under the symmetric-design condition, tourists’ typicality perception of ICH souvenirs positively mediated the main relationship, while under the asymmetric-design condition, tourists’ novelty perception had a negative mediating effect. The moderated mediation effects were in accord with hypotheses to some extent; at a relatively high level of authenticity perception (above mean value), the indirect effect of symmetry on aesthetic pleasure *via* typicality perception increased as authenticity perception rose; at a relatively low level of authenticity perception (under mean value), the indirect effect of symmetry on aesthetic pleasure *via* novelty perception declined as authenticity perception rose. This study identified critical factors influencing tourists’ aesthetic pleasure with ICH souvenirs, and it revealed the internal influencing mechanisms and moderating effects under different design conditions. These findings give some insights to ICH practitioners for using souvenir design to improve tourists’ aesthetic pleasure.

## Introduction

Souvenirs function as mementos of tourism experiences at particular destinations and have become an essential component of tourism (Gordon, 1986). Compared to acquiring other objects, possessing souvenirs helps to maintain memories as well as expressions of individuality and identity (Decrop and Masset, 2014). As a metonymic and metaphoric act, souvenir purchasing is a part of the ritual of tourism, closely connecting with the understanding and stereotypical perceptions of local culture (Littrell et al., 1993; Anastasiadou and Vettese, 2021). In order to meet the expanding demand for souvenirs with local features, increasing amounts of intangible cultural heritage (ICH) are developed as souvenirs based on their representativeness and locality (Soukhathammavonga and Park, 2019). Tourism research on souvenirs has explored this field from the perspective of tourists’ motivation (Swanson and Horridge, 2006), perception (Littrell et al., 1993; Peters, 2011; Torabian and Arai, 2016), and behavior intentions (Kim and Littrell, 1999; Lin and Wang, 2012; Altintzoglou et al., 2016), in addition to the benefits of ICH materialized (Soukhathammavonga and Park, 2019), but it has neglected the aesthetic aspect. Tribe (2009) indicates that there is close connection between tourism and aesthetics, and studies have acknowledged the crucial value of aesthetic in tourism experience (Oh et al., 2007; Alegre and Garau, 2010; Dans and González, 2019). As experiencing beauty is one of the main attractions of tourism (Maitland and Smith, 2009; Kirillova et al., 2014), as well as the essential role of the aesthetic in heritage tourism (Trinh and Ryan, 2016), it is reasonable and worthwhile to examine the question: *how does ICH souvenir design influence tourists’ aesthetic pleasure?*

Beauty has increasingly become a core attribute of objects in modern society (Candi et al., 2017; Castagna et al., 2021), and many studies have considered how aesthetic pleasure derives from everyday objects design (Hekkert et al., 2003; Blijlevens et al., 2012). However, little is known about how tourists’ aesthetic pleasure is influenced by tourist product design. To fill the research gap, this paper aims to explore the impact of symmetry design of ICH souvenirs on tourists’ aesthetic pleasure. There are two chief reasons for considering symmetry design: First, the impact of symmetry on aesthetic evaluation has been widely recognized (Little et al., 2007; Tinio and Leder, 2009; Bode et al., 2017; Leder et al., 2019; Gartus et al., 2020). Second, symmetrical patterns have often been used in ICH to display an artistic nature and achieve a prefect rhythm (Wang, 2019). Moreover, as an independent variable, the impacts of typicality perception and novelty perception on aesthetic response have been examined (Pedersen, 1986; Bornstein, 1989; Hekkert and van Wieringen, 1990; Repp, 1997; Veryzer and Hutchinson, 1998; Reber et al., 2004), but there is no consensus among the research findings. In this study, we explore the mediating role of typicality perception and novelty perception, expanding the effects of these variables in the aesthetic process. ICH souvenirs can be handmade and perceived as artistically superior and exemplary reflections of local culture (Cave and Buda, 2013; Hitchcock, 2013). Commercial, mass-produced ICH is quite common and includes cheap, meaningless, replicated things. Therefore, the concept of authenticity has received attention in the academic literature to address the problem of over-commercialization (Kolar and Zabkar, 2010; Fu et al., 2018; Anastasiadou and Vettese, 2021). In this study, we use authenticity perception as a moderating variable to test how the impact of symmetry design of ICH souvenirs on tourists’ aesthetic pleasure changes under different circumstances, as well as the mediating effects of typicality perception and novelty perception.

With plenty of ICH transformed into commodity products, it is very necessary to analyze what kind of ICH souvenir can bring pleasurable experiences to tourists. This paper employs two lab-based experiments to test the direct effects of symmetry design on ICH souvenirs and the contrasting mediation paths under symmetric and asymmetric-design conditions. This analysis distinguishes this study from previous studies, which have only concentrated on one situation. Furthermore, the paper provides insight into entirely different moderating effects of authenticity perception on the psychological mechanisms underlying the positive relationship between symmetry design and tourists’ aesthetic pleasure, which advance the understanding of how ICH souvenir design might be used to help tourism practitioners and ICH inheritors develop both popular and valuable souvenirs.

The following section reviews the literature on souvenirs, ICH, and symmetry design. Accordingly, five hypotheses are proposed, comprising the direct effect, mediating effects, and moderated mediating effects. Then, the methodology will be introduced, wherein hypotheses will be tested by two between-subjects design experiments. Data analysis and test results will be reported. Finally, the conclusion, theoretical and management implications, and future research will be discussed.

## Literature review and hypotheses development

### Souvenirs and ICH

The common understanding of souvenirs is that they help one to “remember,” or to be reminded of, special times and/or locales (Gordon, 1986). For tourists, souvenirs work as a linkage between daily life and distant places (Morgan and Pritchard, 2005; Ramsay, 2009). As material objects that can be taken away from a place, souvenirs make tangible the intangible encounters that tourists experience (Haldrup, 2017; Li and Ryan, 2018; Anastasiadoua and Vettese, 2019). Souvenirs also refer to a symbol, a representation of personal significance, which can strengthen identities and memories of other cultures (Littrell et al., 1994; Smith and Reid, 1994; Wilkins, 2011; Torabian and Arai, 2016). Gordon (1986) classified souvenirs in tourism in five categories: (1) Pictorial images, such as postcards, which are static reminders that help tourists record ephemeral events; (2) piece-of-rock souvenirs, such as shells, which are saved from a natural environment; (3) symbolic shorthand souvenirs, such as a miniature Sphinx or pyramid from Egypt, which are usually manufactured; (4) “markers” as souvenirs, such as an “I Love NY” T-shirt, which are objects inscribed with specific words or signs; and (5) local product souvenirs, such as indigenous foods, which are only available in a local community. The consumption of souvenirs is an essential element of tourist experience and generates momentous revenue streams for tourism destinations and merchants (Swanson and Timothy, 2012; Horodyski and Gândara, 2016; Kong and Chang, 2016; Jin et al., 2017). According to the model of the five types of souvenirs, production relies on locality (Soukhathammavonga and Park, 2019), which is a distinguishing feature of ICH.

ICH is the cultural manifestation and wealth of knowledge and skills transmitted from generation to generation (UNWTO, 2012). Locality refers to that the development of practices, representations, and expressions is relevant to a particular place and environment where a community lives (Arizpe and Amescua, 2013). UNESCO World Heritage Centre (2003) proposes five domains of ICH: performing arts, oral traditions and expressions, knowledge and practices relative to nature and the universe, traditional craftsmanship, and community practices and rituals. Traditional craftsmanship is the likely domain for souvenir development. There are numerous kinds of traditional craftsmanship, such as clothing or costumes, jewelry, storage containers, decorative art, instruments, utensils, and toys. The purposes of these traditional craftsmanship are diversified; some are only used for particular festivals and rituals, while some are used for amusement, education, or other daily needs. Therefore, souvenirs that are developed based on ICH, especially based on the traditional craftsmanship that is a tangible manifestation of ICH, have very high marketability.

In this study, ICH souvenirs are defined as products that are related to the cultures that are transmitted from generation to generation, and recognized as part of expression of ICH by communities and groups. According to the Safeguarding of the Intangible Cultural Heritage (UNESCO World Heritage Centre, 2003), traditional craftsmanship relies on hand production, but that is not a pragmatic method for souvenir production, because it takes too much time, and the output is too small, compared to mass tourism, while prices are usually much higher than with mass- production souvenirs. As mass production is necessary to some extent, materialized ICH in the form of souvenir results in commodification of local culture (Soukhathammavonga and Park, 2019). Handmade souvenirs are considered genuine artistic works, whereas mass-produced souvenirs are regarded as cheap and inauthentic commercial items (Thompson et al., 2012). Although the commercial activities of ICH souvenir production have attracted criticism about commercialism and cultural deterioration, studies about the souvenirs developed based on heritage only concentrate on tourists’ perceptions (Littrell et al., 1993; Peters, 2011; Torabian and Arai, 2016), motivations (Swanson and Horridge, 2006) and purchasing behaviors (Wilkins, 2011; Lin and Wang, 2012; Altintzoglou et al., 2016). They seldom concern the aesthetic aspect of ICH souvenirs, which has close connections with cultural appreciation under the inevitable commodification process. Therefore, this study explores how the design of ICH souvenirs (symmetry vs. asymmetry) influences tourists’ aesthetic pleasure.

### Aesthetic in tourism experience

Aesthetic is one of basic values to human being (Shusterman and Tomlin, 2008). In tourism experience, aesthetic is the core element, which contributes to tourists’ motivations, perceptions, satisfaction, and intentions to revisit or recommend (Hosany and Witham, 2009; Alegre and Garau, 2010; Kirillova and Lehto, 2015). Since the beauty of nature-based destinations is vital for tourism development (Yoon and Uysal, 2005), Breiby (2014) investigates the influence of aesthetic dimensions in nature-based tourism context and Kirillova et al. (2014) further reveal dimensions of aesthetic judgment in both nature-based and urban destinations. Le et al. (2019) find that tourists’ perceived beauty is sensitive to environmental changes. Zhang and Xu (2020) demonstrate the impact of natural aesthetic experiences on tourists’ loyalty. For tourism amenities, such as hotels, industry specialists have pointed out the trend and importance of aesthetic expressivity (Strannegård and Strannegård, 2012).

In cultural heritage tourism, Trinh and Ryan (2016) suggest that aesthetic experience has been neglected. As aesthetics and culture are inherent component of presentation, aesthetic plays an critical role in heritage tourism (Halewood and Hannam, 2001). Furthermore, consumers become more interested in the appearance of their possessions and visually attractiveness of products (such as souvenirs) significantly influence people’s perceptions and behaviors (Weaver, 2009). Although literatures have recognized the importance of aesthetic in tourism experience (Oh et al., 2007; Alegre and Garau, 2010; Dans and González, 2019), there is still an unanticipated insufficiency of empirical research related to linkage between ICH souvenir design and aesthetic pleasure.

### Symmetry design and tourists’ aesthetic pleasure

In the *Oxford English Dictionary*, “symmetry” is defined as “the quality of being made up of exactly similar parts facing each other or around an axis.” There are three basic types of symmetry: translational, rotational, and mirror symmetry (Wagemans, 1995). As mirror symmetry can be detected more easily than other two types, it is very common in pattern design (Palmer and Hemenway, 1978; Bertamini et al., 2002) as well as in ICH souvenir design. There is a consensus that symmetry is preferred over asymmetry in various domains, such as human faces and visual patterns (Shepherd and Bar, 2011). The theory of beauty is developed in facial attractiveness study, which proposes that the perception of beauty positively relates to facial symmetry (Rhodes et al., 1998). Based on this theory, the impact of symmetry on aesthetic evaluation is positive and robust among various cultures, genders, and age groups (Little et al., 2007; Tinio and Leder, 2009; Bode et al., 2017; Leder et al., 2019; Gartus et al., 2020). Aesthetic evaluation refers to understanding of beautiful things and giving estimation of beauty. Although the objectivist perspective, subjectivist perspective, and interactionist perspective of beauty reflect the differing views about what beauty is, it is undeniable that beauty relates to the beholder’s cognitive and affective reactions toward an object (Tatarkiewicz, 1970).

Based on the connotation of beauty, aesthetic pleasure can be defined as the pleasurable perceptions that derive from dealing with an object for its own sake (Dutton, 2009; Blijlevens et al., 2017). People can find aesthetic pleasure from many stimuli, such as a sunset, a painting, or a piece of music. Nowadays, objects are deliberately designed to bring aesthetic pleasure (Postrel, 2003). In the design elements, symmetry has been used frequently to improve beauty perception. As the intrinsic feature of beauty is immediate joy without intermediate reasoning (Maritain, 1966), we suggest that symmetry positively influences aesthetic pleasure. Moreover, psychological research has demonstrated that the innate preference for symmetry results from more fluent processing than asymmetry (Garner, 1974; Reber, 2002). Reber et al. (2004) propose that the more fluent the process of an object, and the more positive aesthetic response arising, the more positive impact on aesthetic pleasure. As symmetry design has been widely used in ICH souvenir development, the following hypothesis is proposed:

*H1*: Symmetry design of ICH souvenir positively influences tourists’ aesthetic pleasure.

### The mediation effect of typicality perception

“Typicality” means that an object shows the most usual characteristics of a particular type of product (Hekkert, 2014). Typicality perception is related to category schema (Moreau et al., 2001); that is, if an object is deemed to be a good example of a particular category, typicality perception is high. In order to form categories, people need to experience an object repeatedly, which is inherently typified (Hekkert, 2014). When a new object is experienced, people do not straightforwardly compare it with a typical item of a category but involve both sensory and motor functions to assess its typicality (Barsalou et al., 2003; Gallese and Lakoff, 2005). As a perceptual cue, symmetry plays a vital role in this assessment process. A symmetrical pattern usually involves less information than an asymmetrical pattern and thus is easier to process (Garner, 1974; Reber et al., 2004). Accordingly, an object with symmetrical design is more likely to be categorized than an asymmetrical one.

The preference-for-prototypes theory suggests that an object is affectively processed within its category rather than being unique, and that the perceiver’s aesthetic satisfaction is influenced by the extent to which the object is conceived typical of a category (Whitfield and Slatter, 1979). Proximity to category prototype would evoke favorable aesthetic responses. The higher an object’s typicality, the more it will be aesthetically preferred (Hekkert et al., 2003). Many empirical studies have demonstrated the positive relationship between prototype and aesthetic preference among various human artifacts (Pedersen, 1986; Hekkert and van Wieringen, 1990; Repp, 1997; Veryzer and Hutchinson, 1998). Furthermore, several studies have verified the positive impact of typicality on aesthetic pleasure (Blijlevens et al., 2014a, 2017). As symmetry can be expected to have a positive impact on typicality and aesthetic pleasure, and as typicality has a positive impact on aesthetic pleasure, the following hypothesis is proposed:

*H2*: When the design of an ICH souvenir is symmetrical, a typicality perception of ICH souvenir positively mediates the relationship between symmetry and tourist’s aesthetic pleasure.

### The mediation effect of novelty perception

Novelty perception is induced by a new, different, and interesting object that is unusual or novel (Mukherjee and Hoyer, 2001; Mugge and Dahl, 2013). Prior research has found that an asymmetrical design pattern creates a higher level of arousal (Berlyne, 1971; Locher and Nodine, 1989). Meanwhile, asymmetry pattern can increase visual complexity, since it contains more visual information than a symmetrical one (Pieters et al., 2010). As asymmetric patterns can give rise to excitement and uniqueness (Krupinski and Locher, 1988), we suggest that asymmetry in design leads to novelty perception; in other words, symmetry has a negative impact on novelty perception.

People prefer familiar choices, but not novel choices. Because of evolutionary advantages, it is safe to choose a familiar object but not a strange one that is potentially harmful and threatening (Bornstein, 1989). Moreover, novel stimuli elicit more attention than familiar or typical stimuli, which can lead to less-fluent processing (Reber et al., 2004). Previous studies have suggested increasing exposure times to novel items to improve perceptual fluency (Whittlesea and Price, 2001). High fluency is subjectively experienced as pleasure (Reber et al., 2004), which is why typicality contributes to aesthetic pleasure. Accordingly, the aesthetic consequences of novelty and typicality would be opposite, and it has been verified that the relationship between novelty and typicality is negative (Whitfield and Slatter, 1979). As symmetry can be expected to have a negative impact on novelty, and novelty has negative impact on aesthetic pleasure, while it is presumed that symmetry positively influences aesthetic pleasure, the following hypothesis is proposed:

*H3*: When the design of an ICH souvenir is asymmetric, novelty perception of an ICH souvenir negatively mediates the relationship between symmetry and tourists’ aesthetic pleasure.

### The moderating effect of authenticity perception

The original concept of authenticity is borrowed from studies of museums, and, in tourism studies, the genesis of authenticity is the debate made by Boorstin (1964) and MacCannell (1973). Boorstin (1964) proposed “Pseudo-Events” to describe mass tourism, while MacCannell (1973) believed that tourists seek authenticity to help them get away from their daily lives. In the development of this dynamic concept, it has been recognized that authenticity has four theoretical perspectives: objective, constructive, existential, and postmodern (Wang, 1999). Within these theoretical perspectives, authenticity research concerns various analytical points, from objects to experiences (Rickly-Boyd, 2012). Souvenir authenticity has received growing attention from scholars and has become an important topic in authenticity research (Swanson, 2013). The positive impact of souvenir authenticity on behavioral intention has been verified (Kolar and Zabkar, 2010; Fu et al., 2018). Anastasiadou and Vettese (2021) reveal how additive manufacturing influences visitors’ perceptions of souvenir authenticity.

“Authentic souvenirs” are products developed based on the representativeness of a particular culture, heritage, place identity, event, or activity in a specific destination (Soukhathammavonga and Park, 2019). They are symbolic markers of a community’s ethnicity or cultural identity (Cohen, 1988). The authenticity perception of ICH souvenirs can be deemed as the impressions of tourists regarding the cultural and historical genuineness and integrity of souvenirs and their attributes (Littrell et al., 1993). When authenticity perception is high, the ICH souvenir represents local community better than the ICH souvenir with low authenticity perception. Under that condition, the effect of symmetry design is expanded, because the value of a typical cultural symbol is perceived by a tourist deeply. Therefore, the following hypothesis is proposed:

*H4*: Compared to ICH souvenirs with low authenticity perception, the effect of symmetry on aesthetic pleasure *via* typicality perception increases for ICH souvenirs with high authenticity perception.

Many reasons lead to the gradual decline of ICH souvenir authenticity perception, including ease of mass production, original design disappearing, catering to visitor preference, and using lighter materials for transportation efficiency (Timothy, 2005; Hitchcock, 2013). The increasing industrialization and commodification of tourism may lead to an ICH souvenir losing its sacredness, cultural meaning, and authenticity (Swanson, 2013). Under this condition, the novelty perception is impaired, because of the homogenous product resulting from over-commercialization. As asymmetry endows objects with peculiarity features, among low-authenticity ICH souvenirs, an asymmetrically designed souvenir may bring more novelty perception than a symmetrical one. Consequently, compared to an ICH souvenir with high authenticity perception, the negative effect of symmetry would be higher under low-authenticity-perception conditions. Therefore, the following hypothesis is proposed:

*H5*: Compared to ICH souvenirs with low authenticity perception, the effect of symmetry on aesthetic pleasure *via* novelty perception declines for ICH souvenirs with high authenticity perception.

**Figure 1:**
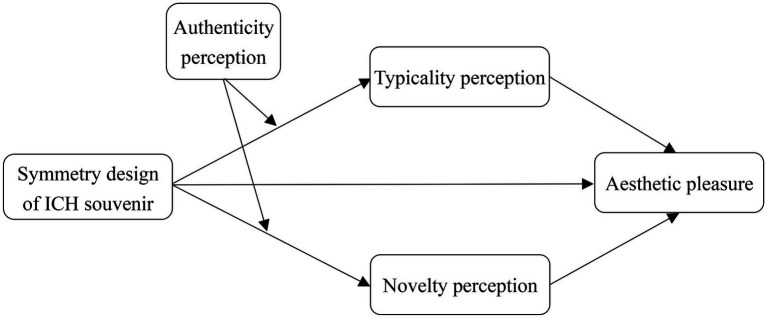
The conceptual model.

The conceptual model proposed by this study is illustrated in Figure 1.

## Methodology and results

### Study 1

#### Purpose

Study 1 tested whether the design of ICH souvenir (symmetry vs. asymmetry) influenced tourists’ aesthetic pleasure. Meanwhile, Study 1 also tested the mediating effect of typicality perception and novelty perception of ICH souvenirs to explore the internal influencing mechanism for different design styles (symmetry vs. asymmetry).

### Method

#### Design and participants

A lab-based experiment was designed to manipulate the design of ICH souvenirs (symmetry vs. asymmetry). College-student participants were randomly assigned to a symmetry or asymmetry condition. A total of 112 valid questionnaires were obtained (41.1% male, 58.9% female; *M*_age_ = 19.13, SD = 0.895).

#### Procedure

**Figure 2:**
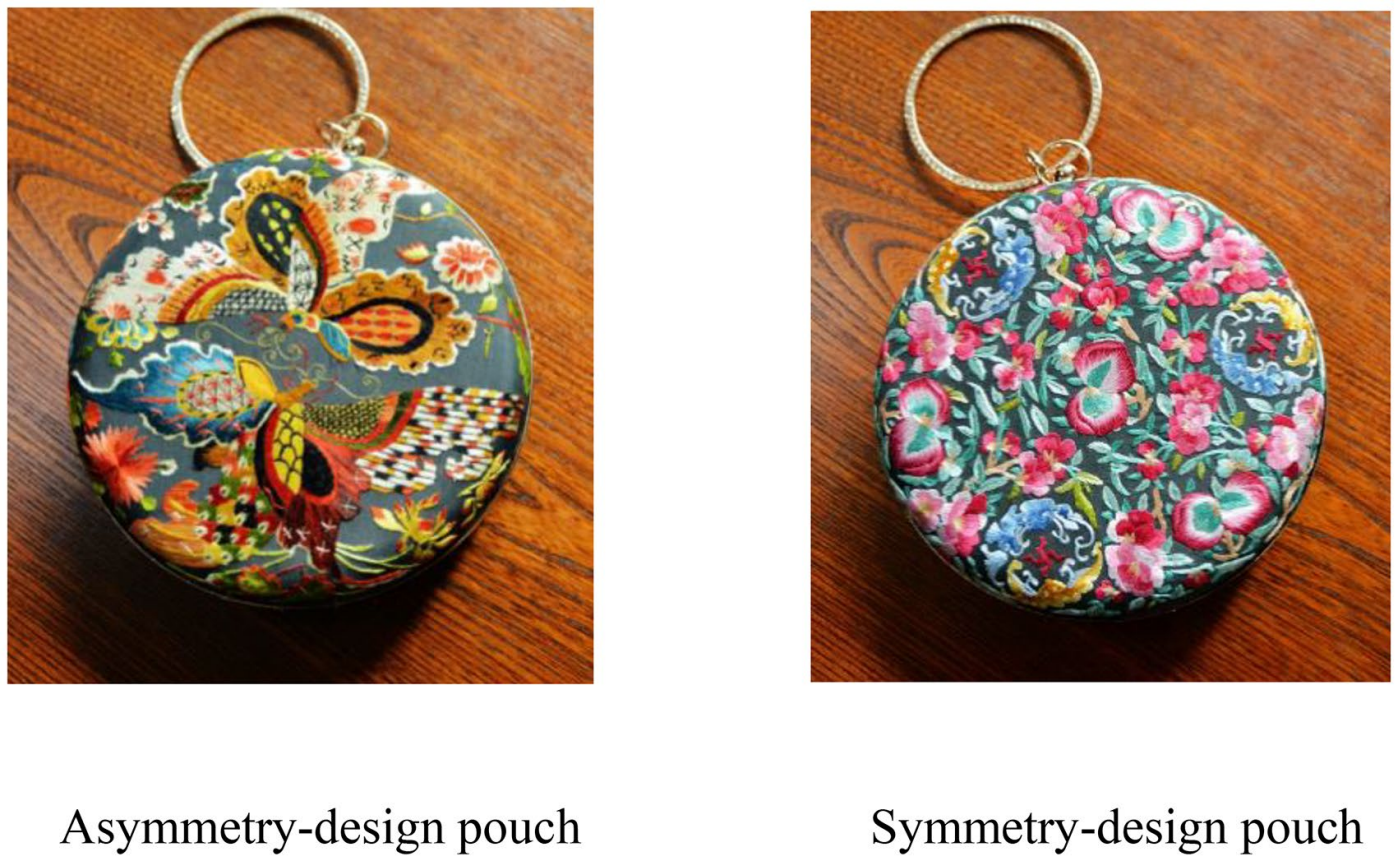
Picture of ICH souvenir stimuli used in Study 1.

Participants were first required to read an introduction of the ICH souvenir. A pouch based on Suzhou embroidery (a very famous kind of intangible cultural heritage) was chosen as a stimulus. A symmetry design picture and an asymmetry design picture of the pouch were adopted from Shatangxiu, who is an ICH souvenir designer. Participants then looked at a symmetrically designed pouch or an asymmetrically designed pouch developed based on Suzhou embroidery (see Figure 2) and answered the following questionnaire.

#### Measures

All variables were measured by scales suggested by previous literatures. For Chinese respondents, a back-translation procedure (Brislin, 1986) was performed to improve the accuracy of measurement.

A seven-point Likert scale was used for indicating levels of agreement, ranging from 1 (strongly disagree) to 7 (strongly agree). For each scale with several items, items were averaged into a single measure.

Symmetry was measured with two items adopted from Bettels and Wiedmann (2019). Typicality perception was measured with three items suggested by Berghman and Hekkert (2017) and Blijlevens et al. (2017). Novelty perception was measured with four items adopted from Berghman and Hekkert (2017) and Blijlevens et al. (2017). Aesthetic pleasure was measured with five items from Blijlevens et al. (2014b) and da Silva et al. (2016). The specific items are shown in Table 1. Item loadings of all variables exceeded the cut-off level of 0.60 recommended by Hair et al.’s (2010). Meanwhile the average variance extracted (AVE) value all surpassed the cut-off level of 0.50 recommended by Fornell and Larcker (1981). Thus, the convergent validity of the scale was confirmed. As presented in Table 1, the Cronbach’s alpha value of all variables were above the 0.7 threshold, and the composite reliability (CR) scores were all above the 0.7 threshold (Hair et al., 2010). The square roots of AVE values for each variable were greater than their inter-correlations, which means that the discriminant validity of the scales was satisfied (see Table 2). Therefore, the scales had good validity and reliability. According to Harman’s one-factor test and principal component factor analysis, the highest variance was 34.60%, which was lower than 50%. Common method bias was not a problem in study 1.

**Table 1:** Reliability and convergent validity.

Construct/Item	Loading	AVE	CR	Cronbach’s Alpha
*Symmetry (Sym)*		0.764	0.866	0.737
Sym1 The Suzhou embroidery pattern of the pouch is symmetrical.	0.911			
Sym2 The Suzhou embroidery pattern of the pouch has symmetrical features.	0.836			
*Typicality Perception (TP)*		0.661	0.854	0.775
TP1 This is a typical pouch.	0.854			
TP2 This design is common for a pouch.	0.812			
TP3 This is a standard design for a pouch.	0.771			
*Novelty Perception (NP)*		0.688	0.898	0.846
NP1 This is a novel pouch.	0.788			
NP2 This design is original for a pouch.	0.768			
NP3 This is a new example of a pouch.	0.862			
NP4 This design is innovative for a pouch.	0.893			
*Aesthetic Pleasure (AP)*		0.691	0.918	0.887
AP1 This is a beautiful pouch.	0.813			
AP2 This is an attractive pouch.	0.859			
AP3 I like to look at this pouch.	0.822			
AP4 It is nice to see this pouch.	0.820			
AP5 It is pleasing to see this pouch.	0.841			

**Table 2:** Variables correlations and square roots of AVEs.

	1	2	3	4
1 Symmetry	**0.874**			
2 Typicality Perception	0.156	**0.813**		
3 Novelty Perception	0.260	−0.049	**0.829**	
4 Aesthetic Pleasure	0.725	0.315	0.114	**0.831**

Boldface numbers on the diagonal indicate the square roots of the AVE values for variables.

### Results

#### Manipulation check

An independent-samples T test showed that there was a significant difference between the means of the symmetry group and asymmetry group (*M*_symmetry_ = 5.52, *M*_asymmetry_ = 3.12, *p* < 0.001). A one-way ANOVA revealed that the manipulation of ICH souvenir symmetry design had a significant effect on symmetry assessment [*F*(1, 110) = 159.99, *p* < 0.001]. Thus, the manipulation was effective.

#### Hypothesis tests

H1 suggested that the symmetry of an ICH souvenir positively affects tourists’ aesthetic pleasure. Consistent with the hypotheses, the one-way ANOVA showed that the main impact of symmetry on aesthetic pleasure was significant [*M*_high_ = 5.88, *M*_low_ = 4.27, *F*(1, 110) = 12.30, *p* < 0.001]. Thus, H1 was supported.

H2 suggested that tourist’s typicality perception of ICH souvenir would positively mediate the effect of symmetry on aesthetic pleasure when the ICH souvenir was designed symmetrically, while novelty perception would negatively mediate the above-mentioned effect when the design of an ICH souvenir is asymmetric. To test the different mediation effects, a subgroup approach suggested by Edwards and Lambert (2007) was selected. Multiple regression analysis was conducted by Hayes’s (2018) PROCESS (Model 4) to test the mediating effect of typicality perception in the symmetry design group, and novelty perception in the asymmetry group, using a bias-corrected bootstrap procedure (95% confidence intervals, 5,000 bootstrap samples).

In the symmetry design group, the direct effects of symmetry on typicality perception (*β* = 0.433, LLCI = 0.210, ULCI = 0.656, not including 0) and aesthetic pleasure (*β* = 0.252, LLCI = 0.009, ULCI = 0.494, not including 0) were significant. The indirect effect of symmetry on aesthetic pleasure (*β* = 0.096, LLCI = 0.005, ULCI = 0.235, not including 0) was also significant, but the indirect effect of symmetry *via* novelty perception on aesthetic pleasure was not significant (LLCI = −0.127, ULCI = 0.059, including 0). Thus, typicality perception partially mediated the effect of symmetry on aesthetic pleasure. These findings provide support for H1 and H2.

In the asymmetry design group, the direct effects of symmetry on novelty perception (*β* = −0.376, LLCI = −0.633, ULCI = −0.120, not including 0) and aesthetic pleasure (*β* = 0.524, LLCI = 0.167, ULCI = 0.880, not including 0) were significant. The effect of novelty perception on aesthetic pleasure (*β* = −0.389, LLCI = −0.760, ULCI = −0.018, not including 0) was significant. The indirect effect of symmetry on aesthetic pleasure (*β* = 0.146, LLCI = 0.012, ULCI = 0.363, not including 0) was also significant. The indirect effect of symmetry *via* typicality perception on aesthetic pleasure was not significant (LLCI = −0.011, ULCI = 0.496, including 0). Thus, novelty perception partially mediated the effect of symmetry on aesthetic pleasure. These findings provide support for H1 and H3.

### Discussion

The findings showed that symmetry of ICH souvenir design had a positive impact on tourists’ aesthetic pleasure. Under the symmetric-design condition, tourists’ typicality perception of ICH souvenirs positively mediated the main relationship, while under the asymmetric-design condition, tourists’ novelty perception had a negative mediating effect. As authenticity is a crucial feature in souvenir development based on ICH, this study further analyzes the moderating role of tourists’ authenticity perception of ICH souvenirs in Study 2.

### Study 2

#### Purpose

Study 2 re-examined the relationship between ICH souvenir symmetry and tourists’ aesthetic pleasure, as well as the distinct mediating effect of typicality perception and novelty perception under symmetry or asymmetry ICH souvenir design conditions. Meanwhile, Study 2 tested the moderating effect of authenticity perception, especially the moderated mediation effect under different design conditions (symmetry vs. asymmetry).

### Method

#### Design and participants

A lab-based experiment was designed to manipulate the design of ICH souvenirs (symmetry vs. asymmetry) and the authenticity degree (high vs. low) of ICH souvenirs. A 2 (symmetry vs. asymmetry) × 2 (authenticity degree: high vs. low) between-subjects study design was employed. Participants were randomly assigned to one of these conditions. A total of 165 valid questionnaires were obtained (47.3% male, 52.7% female; *M*_age_ = 24.4, SD = 7.44).

#### Procedure

**Figure 3:**
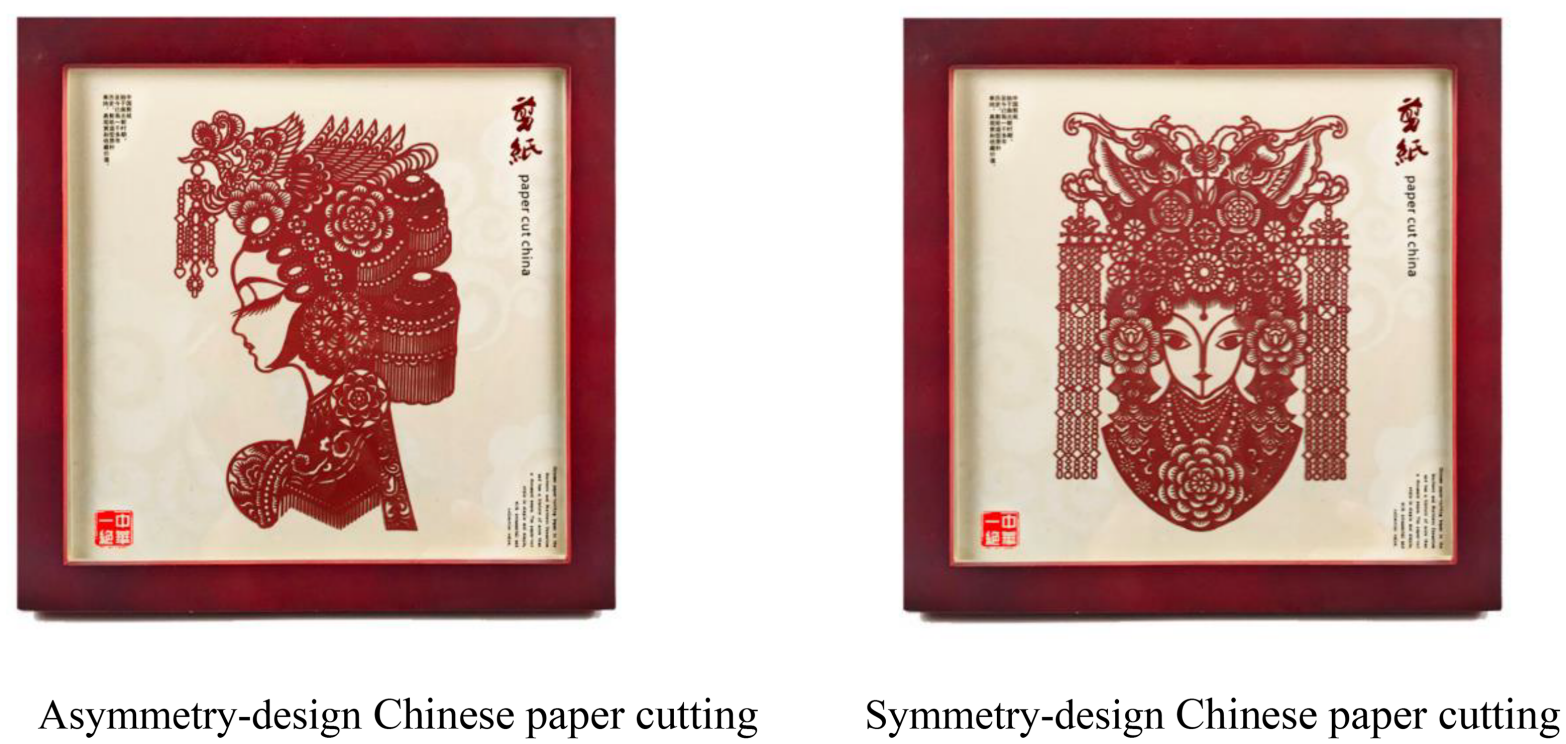
Picture of ICH souvenir stimuli used in Study 2.

Chinese paper cutting, as a very famous kind of intangible cultural heritage, was chosen as a stimulus. Symmetrical picture design and asymmetrical picture design of the Chinese paper cutting were adopted from Tao’s Shadow and Paper Cutting Factory, which is an ICH souvenir designer and manufacturer. Participants were first required to read introductions of the ICH souvenir, which contained the manipulation of the authenticity degree. For a high degree of authenticity, the text was: “Chinese paper cutting is authenticated by historians as state-level intangible cultural heritage. Chinese paper cutting is a kind of folk art with a long history. Please appreciate the following souvenir developed based on the Chinese paper cutting, which is handmade by inheritor of intangible cultural heritage.” For a low degree of authenticity, the text was: “Chinese paper cutting is a kind of intangible cultural heritage. Chinese paper cutting is a unique folk art. Please appreciate the following souvenir developed based on the Chinese paper cutting, which is mass-produced by professional manufactory.” Then participants looked at a symmetrically designed Chinese paper cutting or an asymmetrically designed Chinese paper cutting (see Figure 3) and answered the following questionnaire.

Asymmetry-design Chinese paper cutting Symmetry-design Chinese paper cutting.

#### Measures

In Study 2, the measurements of symmetry, typicality perception, novelty perception, and aesthetic pleasure were the same as in Study 1, except for the object “pouch” which was replaced with Chinese paper cutting. For the measurement of authenticity perception, items were adopted from Ramkissoon and Uysal (2011) and Zhou et al. (2013). These were “This Chinese paper cutting souvenir has a long history,” “This Chinese paper cutting souvenir is handmade by local people,” “This Chinese paper cutting souvenir is representative of local life,” and “This Chinese paper cutting souvenir is authenticated by historians.” A back-translation procedure (Brislin, 1986) was also performed. A seven-point Likert scale was used, ranging from 1 (strongly disagree) to 7 (strongly agree), and items were averaged into a single measure. The coefficient alpha for the measurement scale of authenticity perception was 0.848. The factor loadings (0.852, 0.781, 0.864, 0.827) were all above the threshold. The AVE value of authenticity perception was 0.692, the CR value was 0.899, and the square root of the AVE was 0.832 which was greater than the correlations between variables (−0.317, −0.080, 0.067, −0.163). Therefore, the validity and reliability of the scales was confirmed. In terms of the Harman’s one-factor test, the highest variance was 27.27% which was lower than 50%. Thus, common method bias was not a problem in study 2.

### Results

#### Manipulation check

A two-way ANOVA revealed that the manipulation of ICH souvenir symmetry design had a significant effect on symmetry assessment (*M*_symmetry_ = 5.75, *M*_asymmetry_ = 3.26, *p* < 0.001, *F*(1,161) = 143.32, p < 0.001), and the main effect of ICH authenticity perception manipulation was significant (*M*_high-authenticity perception_ = 5.71, *M*_low-authenticity perception_ = 3.63, p < 0.001, *F*(1,161) = 125.56). Thus, the manipulations were effective.

#### Hypothesis tests

H4 and H5 suggested that there were different moderated mediation effects when authenticity perception of ICH souvenir was high rather than low. A bootstrap method (Hayes, 2018) was used to test the moderated mediation effects in the symmetry group or the asymmetry group, respectively (PROCESS, model 7), with bias-corrected bootstrap procedure (95% confidence intervals, 5,000 bootstrap samples).

In the symmetry design group, the direct effect of symmetry on aesthetic pleasure (*β* = 0.446, LLCI = 0.278, ULCI = 0.614, not including 0) was significant. The interaction effect of symmetry and authenticity perception on typicality perception (*β* = 0.086, LLCI = 0.023, ULCI = 0.170, not including 0) was significant. The effect of typicality perception on aesthetic pleasure (*β* = 0.246, LLCI = 0.041, ULCI = 0.450, not including 0) was significant. In terms of the moderated mediation analysis, the indirect effect of symmetry on aesthetic pleasure *via* typicality perception (LLCI = −0.001, ULCI = 0.075, including 0) was not always significant with the change of authenticity perception level. Yet, results revealed a similar trend with H4: when ICH souvenir authenticity perception was low (average value on authenticity-perception scores), the indirect impact of symmetry on aesthetic pleasure *via* typicality perception (*β* = 0.045, LLCI = 0.001, ULCI = 0.175, not including 0) was lower than the impact (*β* = 0.088, LLCI = 0.001, ULCI = 0.263, not including 0) when the authenticity perception was high (+1 SD on authenticity perception scores). The indirect effect of symmetry on aesthetic pleasure *via* novelty perception (LLCI = −0.033, ULCI = 0.015, including 0) was not significant. Thus, H1 and H2 were supported, and H4 was partially supported.

In the asymmetry design group, the direct effect of symmetry on aesthetic pleasure (*β* = 0.203, LLCI = 0.007, ULCI = 0.399, not including 0) was significant. The interaction effect of symmetry and authenticity perception on novelty perception (*β* = 0.140, LLCI = 0.036, ULCI = 0.243, not including 0) was significant. The effect of novelty perception on aesthetic pleasure (*β* = −0.322, LLCI = −0.635, ULCI = −0.010, not including 0) was significant. In terms of the moderated mediation analysis, the indirect effect of symmetry on aesthetic pleasure *via* novelty perception (LLCI = −0.128, ULCI = 0.0004, including 0) was not always significant with the change of authenticity-perception level. Yet, results revealed a similar trend with H5: when ICH souvenir-authenticity perception was low (−1 SD on authenticity perception scores), the indirect impact of symmetry on aesthetic pleasure *via* novelty perception (*β* = 0.114, LLCI = 0.009, ULCI = 0.294, not including 0) was higher than the impact (*β* = 0.056, LLCI = 0.005, ULCI = 0.164, not including 0) when the authenticity perception was high (average value on authenticity-perception scores). The indirect effect of symmetry on aesthetic pleasure *via* typicality perception (LLCI = −0.018, ULCI = 0.056, including 0) was not significant. Thus, H1 and H3 were supported, and H5 was partially supported.

### Discussion

The moderated mediation effects were in accord with hypotheses to some extent; at a relatively high level of authenticity perception (above mean value), the indirect effect of symmetry on aesthetic pleasure *via* typicality perception increased as authenticity perception rose; at a relatively low level of authenticity perception (under mean value), the indirect effect of symmetry on aesthetic pleasure *via* novelty perception declined as authenticity perception rose. One reason for the partial support may because of the optimal stimulation level theory; that is, the stimulus level is different on objects in the levels (Mcalister and Pessemier, 1982). Thus, more and different experiment stimuli and descriptions should be used to manipulate the authenticity perception.

## Conclusion and implications

### Conclusion

Tourist aesthetic pleasure contributes to both memorable tourism experience and heritage tourism development. The results of Study 1 showed that symmetry design of ICH souvenir had a positive impact on tourists’ aesthetic pleasure. In terms of mediation effect, when the design of ICH souvenir is symmetric, tourists’ typicality perception of ICH souvenir positively mediates the relationship between symmetry and aesthetic pleasure, whereas novelty perception negatively mediates the main effect when the design of an ICH souvenir is asymmetric. Study 2 further explored the moderating effect of authenticity perception of ICH souvenirs, partially verifying a moderated mediation effect. Under symmetry design conditions, the indirect effect of symmetry on aesthetic pleasure *via* typicality perception increased with the rising of authenticity perception from mean value to +1 SD on authenticity-perception scores. Under asymmetry-design conditions, the indirect effect of symmetry on aesthetic pleasure *via* novelty perception declined with the falling of authenticity perception from mean value to −1 SD on authenticity-perception scores. The results of the two experiments (Study 1 and Study 2) confirmed the prediction that the underlying psychological process of the impacts of symmetry design or asymmetry design on aesthetic pleasure were different, as well as the moderating role of authenticity perception.

### Theoretical implications

This paper makes three initial contributions to souvenir aesthetic literature. First, according to the theory of beauty, extant studies have widely attended to the positive of symmetry on aesthetic evaluation varying by cultures, groups, contexts and stimuli (Little et al., 2007; Tinio and Leder, 2009; Bode et al., 2017; Leder et al., 2019; Gartus et al., 2020). This study extends the impact of symmetry design from appraisal research to emotion research, which broaden the scope of research on symmetry and aesthetic response. In line with the extant literature, this study further verifies the positive effect of symmetrical design of ICH souvenir priming on aesthetic pleasure. Moreover, this study bridges the research gap by exploring how to improve tourists’ aesthetic pleasure toward ICH souvenirs, which is seldom considered by souvenir studies. In souvenir research, tourists’ behavior intention has attracted much more attention than aesthetic response (Wilkins, 2011; Lin and Wang, 2012; Altintzoglou et al., 2016). As appreciation of beauty is a vital motivation of tourism, the findings of this study contribute to souvenir research in the matters of aesthetic experience.

Second, this study reveals the internal influencing mechanism of the relationship between symmetry design and aesthetic pleasure to answer the research question, including the positive mediation effect of typicality perception and the negative mediation effect of novelty perception. By uncovering these two mediation paths, this study distinguishes itself from prior research, which augments the theories of the mechanism behind aesthetic response. This study initially provides reasonable explanations about why symmetry design can positively influence aesthetic pleasure, while prior research has only demonstrated the direct effect.

Third, this study also bounds the effect of symmetry design by unfolding the moderating role of authenticity perception to a certain degree. In heritage tourism development research, authenticity has always been an important concept to balance heritage preservation and commercialization (Cohen, 1988). This study demonstrates the amplified effect of authenticity perception in the ICH souvenir symmetry design context and the restricted effect of authenticity perception in the ICH souvenir asymmetry design context, which provides evidence to prove that cooperating with the symbolic significance of authenticity, the effect of symmetry design of ICH souvenirs become prominently in the forming of tourists’ aesthetic pleasure.

### Management implications

This study suggests that promoting symmetrical design of ICH souvenirs would enhance tourists’ aesthetic pleasure. For ICH souvenir developers, symmetry should be deemed as a very important design philosophy. Symmetry in design can be in the form of pattern, shape, color, or any other visual elements. For example, stripe and geometrical shape are easier to design symmetrically than scenery or animal. Furthermore, symmetrical design should be noticed easily by tourists, as prominent cues facilitating aesthetic pleasure. Thus, mirror symmetry is better than translational or rotational symmetry on the drawing board. For ICH inheritors, especially traditional craftsmanship inheritors, on account of the “living” and recreated feature of ICH, symmetry in design should be considered as a component in adapting process to changes of the society, cultural, and environment.

The findings of this study demonstrate the mediating role of typicality perception and novelty perception. Therefore, ICH souvenir designers, exhibitors, and sellers can use some measures to intensify tourists’ typicality perception, but weaken their novelty perception. For example, using visual clues and text interpretations to remind tourists of the category of an ICH souvenir, which helps tourists perceive the typicality; increasing exposure times *via* multiple media; or reducing novelty perception by symmetrical design. At last, as authenticity plays a vital role in the souvenir industry, and this study further examines the moderating role of authenticity perception, several means can be used to improve authenticity perception, including combining handcrafting and mass-production, highlighting originality and avoiding duplication, clarifying the locality and inheritance, and facilitating the communication and connection between tourist and ICH souvenir development.

### Limitations and future research

This study has several limitations that suggest directions for future research. First, in symmetry design manipulation, we only chose symmetric patterns as stimuli; many other design elements and translational symmetry should be used to generalize research findings in the future. Second, although traditional craftsmanship is the most likely kind of ICH to develop into souvenirs, other kinds of ICH may also become souvenirs, and future studies are necessary to explore whether different kinds of ICH souvenirs influence the aesthetic pleasure formation. Nowadays, many new technologies are used in souvenir development, such as 3D-printing, which can display the process of souvenir production in the presence of tourists. In the future, how these real-time productions, interacting with authenticity perception, influence the effect of symmetry design should be further examined.

## Author’s note

YL, Ph.D., Lecturer at School of Management, Tianjin University of Commerce, Tianjin, China. Her research interest focuses on tourism innovation and heritage management. MC, student at School of Management, Tianjin University of Commerce, Tianjin, China. Her research interest focuses on heritage tourism. QW, Ph.D., professor at School of Management, Tianjin University of Commerce, Tianjin, China. His research interest focuses on heritage tourism.

## Data availability statement

The datasets presented in this article are not readily available because the dataset only used for this study. Requests to access the datasets should be directed to liuyuqing@tjcu.edu.cn.

## Author contributions

YL contributed to conceptualization, data analysis, and writing. MC contributed to literature collation, data collection, and writing. QW contributed to project administration and editing. All authors contributed to the article and approved the submitted version.

## Funding

This research project has received financial support from the Tianjin Artistic Science Project Fund granted to Yuqing Liu (No. A18028).

## Conflict of interest

The authors declare that the research was conducted in the absence of any commercial or financial relationships that could be construed as a potential conflict of interest.

## Publisher’s note

All claims expressed in this article are solely those of the authors and do not necessarily represent those of their affiliated organizations, or those of the publisher, the editors and the reviewers. Any product that may be evaluated in this article, or claim that may be made by its manufacturer, is not guaranteed or endorsed by the publisher.
